# Stress Granules Inhibit Coxsackievirus B3-Mediated Cell Death via Reduction of Mitochondrial Reactive Oxygen Species and Viral Extracellular Release

**DOI:** 10.4014/jmb.2210.10027

**Published:** 2023-01-11

**Authors:** Ji-Ye Park, Ok Sarah Shin

**Affiliations:** BK21 Graduate Program, Department of Biomedical Sciences, College of Medicine, Korea University Guro Hospital, Seoul 08308, Republic of Korea

**Keywords:** CVB3, stress granules, G3BP1, apoptosis, mitochondrial reactive oxygen species, small extracellular vesicles

## Abstract

Stress granules (SGs) are cytoplasmic aggregates of RNA-protein complexes that form in response to various cellular stresses and are known to restrict viral access to host translational machinery. However, the underlying molecular mechanisms of SGs during viral infections require further exploration. In this study, we evaluated the effect of SG formation on cellular responses to coxsackievirus B3 (CVB3) infection. Sodium arsenite (AS)-mediated SG formation suppressed cell death induced by tumor necrosis factor-alpha (TNF-a)/cycloheximide (CHX) treatment in HeLa cells, during which G3BP1, an essential SG component, contributed to the modulation of apoptosis pathways. SG formation in response to AS treatment blocked CVB3-mediated cell death, possibly via the reduction of mitochondrial reactive oxygen species. Furthermore, we examined whether AS treatment would affect small extracellular vesicle (sEV) formation and secretion during CVB3 infection and modulate human monocytic cell (THP-1) response. CVB3-enriched sEVs isolated from HeLa cells were able to infect and replicate THP-1 cells without causing cytotoxicity. Interestingly, sEVs from AS-treated HeLa cells inhibited CVB3 replication in THP-1 cells. These findings suggest that SG formation during CVB3 infection modulates cellular response by inhibiting the release of CVB3-enriched sEVs.

## Introduction

Stress granules (SGs) are cytoplasmic compartments composed of 40S ribosomal subunits, translation initiation factors, poly (A)^+^mRNAs, and RNA-binding proteins. Ras-GTPase-activating SH3 domain-binding protein 1 (G3BP1), T cell intracellular antigen-1 (TIA-1), and TIA-1 related proteins (TIAR) are important proteins required for SG formation and stability [1]. Cellular stresses, such as starvation, hypoxia, and viral infection, promote the phosphorylation of eukaryotic translation initiation factor 2 alpha (eIF2α) through activation of various kinases, including general control non-derepressible-2 (GCN2, or eIF2α kinase 4 (EIF2AK4)); pancreatic eIF2α kinase PEK (EIF2AK3)); protein kinase R (PKR) or heme-regulated inhibitor (HRI); or eIF2α kinase 1 (EIF2AK1) [2]. SGs not only affect protein expression by sequestering mRNA into SGs but can directly affect viral replication as viruses hijack host translational machinery for survival [3].

Apoptosis is a key factor in cellular damage caused by coxsackievirus B3 (CVB3) and contributes to symptoms that range from mild to severe systemic inflammation diseases, including viral myocarditis and pancreatitis [4-6]. CVB3 is reported to target SG formation by cleaving or dephosphorylating SG components. For example, SGs are formed early and then actively disassembled late during CVB3 infection and can contribute to antiviral defense mechanisms [7, 8]. The protease activity of CVB3 viral protein 2A can cleave eIF4G, and G3BP1 overexpression in cells can reduce CVB3 protein expression, suggesting the antiviral role of SG components [9]. Although SGs are known to restrict viral access to host translational machinery, the detailed mechanisms behind the role of SGs during CVB3-mediated apoptosis need to be further explored.

Small extracellular vesicles (sEVs) are membrane-enclosed vesicles derived from various cell types. sEVs have been highlighted for their essential role in cell-to-cell communication by their transport of various molecules. EVs are typically categorized in terms of their size and sEVs include EVs that are <150 nm in diameter [10]. Virus-infected cells are known to release sEVs that can transfer viral components, and CVB3, for example, has been reported to release its viral progeny through sEVs, which can then infect nearby cells [11]. Specifically, sEVs can contain receptors for CVB3 entry that enable recipient cell susceptibility to virus infection.

The effect of SG formation on sEV-mediated CVB3 transmission needs further study. Here, we examined whether AS-mediated SG activation suppresses reactive oxygen species (ROS) production and, in addition, if this suppression is essential for inhibiting ROS-dependent apoptosis during CVB3 infection. Our results suggest that SG activation adjusts mitochondrial ROS production in response to TNF-α/CHX treatment or CVB3 infection and modulates cellular response, possibly via inhibiting the extracellular release of CVB3-enriched sEVs.

## Materials and Methods

### Cells, Reagents and Viruses

HeLa cells purchased from American Type Culture Collection (USA) were maintained in DMEM (Corning Mediatech, USA) supplemented with 10% fetal bovine serum (FBS; Corning Mediatech) and 1% penicillin/streptomycin. THP-1 cells were cultured in RPMI 1640 (Corning Mediatech) supplemented with 10% FBS, 2 mM glutamine, 10 mM HEPES, 1 mM sodium pyruvate, 0.05 mM 2-mercaptoethanol, and 1% penicillin/streptomycin. Sodium arsenite (NaAsO2; AS), mitoTEMPO (MT), carbonyl cyanide chlorophenylhydrazone (CCCP), tumor necrosis factor-alpha (TNF-α) and cycloheximide (CHX) were purchased from Sigma-Aldrich (USA).

CVB3 and recombinant CVB3-GFP have been previously described [12]. Viruses were propagated in HeLa cells, and virus titration was conducted using plaque assays as previously described [13].

### Median Tissue Culture Infectious Dose (TCID50) Assay for Virus Titration

HeLa cells were seeded in 96-well plates with 2% FBS in DMEM. The next day, cells were infected with 10-fold serial diluted CVB3-containing supernatants for 2–3 days. After the incubation period, cells were detached and stained with 0.4% Trypan Blue solution (Gibco, USA). The TCID50 was calculated via the Spearman–Kärber method and expressed as TCID50/ml.

### siRNA Transfection

HeLa cells seeded in 12-well plates were transfected with either control scrambled or G3BP1-targeted siRNA (Bioneer, Korea) using RNAiMAX reagent (Invitrogen, USA) according to the manufacturer’s protocol.

### Double Staining Apoptosis Assay

Propidium iodide (PI) (Sigma-Aldrich) is a DNA stain that stains dead cells specifically; Hoechst 33342 (Thermo Fisher Scientific, USA) stain is a cell-permeable nuclear counterstain that emits blue fluorescence when combined with double-stranded DNA. Cells were stained with 5 μg/ml PI for 15 min and subsequently incubated in 5 μg/ml Hoechst stain for 10 min. After double staining, cells were washed with Dulbecco’s phosphate-buffered saline (DPBS). Stained cells were analyzed using the EVOS FL Auto 2 Imaging system (Thermo Fisher Scientific). The % PI-positive cells were counted from at least 500 cells for each condition compiled from at least three to six experiments and calculated by % PI-positive cells/total Hoechst-positive cells.

### Cell Viability Assay

THP-1 cells were seeded in 96-well plates overnight. Samples in triplicate were treated with HeLa cell-derived sEVs at various time points (4, 24, 48, and 72 h). Cell survival was monitored using a Cell Counting Kit-8 Cell Viability Assay (Dojindo Molecular Technologies, Japan) as per the manufacturer’s instructions. Control samples were set to 100% survival, and all other samples were expressed relative to the control.

### Caspase Activity Assay

Activity levels of caspases 3/7 were measured with a Caspase-Glo Assay Kit (Promega, USA) according to the manufacturer’s instructions. HeLa cells seeded in 48-well plates were infected with CVB3 followed by AS treatment. Caspase-Glo reagent (100 μl) was added to each well, mixed, and incubated at room temperature for 1 h. The luminescence of each sample was measured using a Varioskan LUX Multimode microplate reader (Thermo Fisher Scientific).

### Measurement of Reactive Oxygen Species (ROS)

Intracellular ROS was measured using 2¢,7¢-dichlorofluorescin diacetate (DCFDA, Sigma-Aldrich), which is a cell-permeable, non-fluorescent probe that exhibits green fluorescence upon oxidation. Mitochondrial ROS was measured by MitoSOX (Invitrogen), a fluorescent dye specific for mitochondrial superoxide production in cells. Briefly, cells were treated with 10 mM DCFDA or 5 mM MitoSOX in serum-free medium and incubated for 30 min at 37°C. Cells were then washed with DPBS. Cells stained with fluorescent dyes were detected using an EVOS FL Auto 2 Imaging system.

### Measurement of Mitochondrial Membrane Potential (MMP)

MMP was measured by tetramethylrhodamine methyl ester (TMRM, Thermo Fisher Scientific), an indicator dye that localizes in mitochondria and detects mitochondrial membrane depolarization. Cells were treated with 200 nM TMRM in serum-free medium and incubated for 30 min at 37°C. Cells were then washed with DPBS. TMRM fluorescence was measured by a Varioskan LUX Multimode microplate reader. The percentage of change values of relative TMRM fluorescence were calculated based on percentage of sample fluorescence/control fluorescence.

### Oxygen Consumption Rate (OCR) Assay

OCR was measured using a Seahorse XFp Extracellular Flux Analyzer (Seahorse BioSciences, USA) at 37°C as previously described [14, 15]. HeLa cells were seeded in XFp Cell Culture Microplates (Agilent, USA) at 6,000 cells/well overnight before the experiment. On the day of OCR analysis, the cell medium was replaced with basal DMEM medium, and cells were incubated at 37°C in a non-CO_2_ incubator for 1 h. During incubation, the sensor had to be hydrated by loading a sensor cartridge into the ports. After sensor hydration, the plate was placed on the instrument tray according to the manufacturer’s instructions.

### Quantitative Real-Time PCR (qRT-PCR)

Total RNA was extracted using Trizol reagent (Invitrogen), and cDNA was synthesized with a Reverse Transcription System (Promega) according to the manufacturer’s instructions. Power SYBR Green Master Mix (Invitrogen) was used to determine the relative expression of viral genes. Primer sequences were as previously reported [12, 13]. Relative mRNA levels were determined using the comparative ΔΔCt method and were normalized to GAPDH mRNA levels.

### Western Blot Analysis

Cells were harvested and lysed with RIPA buffer (Sigma-Aldrich) containing protease and phosphatase inhibitors (Roche, Switzerland). Protein lysates were separated on a 10–12% SDS-PAGE gel, transferred to polyvinylidene difluoride (PVDF) membranes, and blocked with 5% (w/v) skim milk in Tris-buffered saline (TBS) supplemented with 0.1% Tween-20 (TBS-Tw) for 1 h at room temperature. The PVDF membranes were then incubated with primary antibodies (Cell Signaling Technology, USA: total/cleaved-PARP total/cleaved-caspase-3, total/cleaved-caspase-7, cleaved-caspase-8, total/cleaved-caspase-9, total/phosphorylated-eIF2α; Santa Cruz Biotechnology: G3BP1, TIA-1, GFP, and eIF3η) at 4°C overnight, followed by HRP-conjugated anti-rabbit or anti-mouse IgG secondary antibodies for 1 h at room temperature. Tubulin or β-actin (Abgent, USA) antibody was used as a loading control. The membranes were incubated with Pierce ECL Western Blotting Substrate (ECL Solution Kit; Thermo Fisher Scientific). Band signals were displayed in a Fusion Solo Imaging System (Vilber Lourmat, France). Band intensity measurement was performed by ImageJ software.

### Small Extracellular Vesicle (sEV) Isolation and Analysis

sEV isolation and analysis were conducted as described previously [15, 16]. Briefly, HeLa cells were treated with 0.25 mM AS for 1 h at 37°C and washed with DPBS. Cells were then infected with CVB3-GFP at a multiplicity of infection (MOI) of 1 for 1.5 h. The virus-containing medium was then removed, and cells were incubated in 2%exosome-free FBS (Thermo Scientific)-supplemented medium for 16 h. Following this, the conditioned medium (CM) was collected and concentrated using a Pierce Protein Concentrator PES (Thermo Scientific) according to the protocol. sEV isolation and purification were performed in accordance with the instructions of an Exo-Spin Exosome Purification Kit (Cell Guidance Systems, UK). Isolated sEVs were quantified using nanoparticle tracking analysis with NS300 NanoSight (ATA Scientific, Australia). sEV characterization was demonstrated by Western blot analysis. The sEV samples resuspended in exosome resuspension buffer (Thermo Fisher Scientific) loaded in SDS-PAGE, transferred to PVDF membranes, and incubated with the primary antibodies at 4'C overnight. The primary antibodies of sEV marker were anti-CD9 (Proteintech, USA), anti-ALIX (Abcam, UK) and anti-CD63 (Santacruz Biotechonlogy). Anti-Calnexin (Proteintech), ER marker, was used as a negative marker. After incubation with HRP-conjugated anti-rabbit or anti-mouse IgG secondary antibodies, the proteins expression was observed by Fusion Solo Imaging System.

### Statistical Analysis

Statistical comparisons were performed using an unpaired two-tailed Student’s *t*-test or the Mann–Whitney test (Graphpad Software, USA). *p* < 0.05 was considered statistically significant.

## Results

### The Formation of Stress Granules Inhibits Apoptosis

**Fig. 1:**
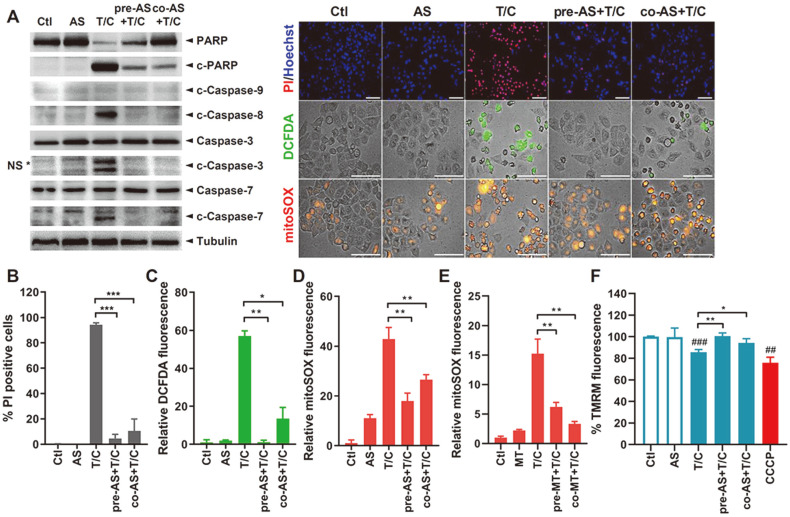
Stress granules (SGs) inhibit TNF-α/CHX-mediated cell death. (**A**) HeLa cells were treated with control (Ctl), 0.25 mM sodium arsenite (AS), TNF-α and cycloheximide (T/C), 0.25 mM sodium arsenite (AS) 1 h prior to stimulation with T/C (pre-AS+T/C), or co-stimulated (co-AS+T/C) for 3 h. The cleavage of caspase-3, caspase-7, and caspase-8, caspase-9, and PARP protein level is shown by Western blot analysis. Images are representatives of three independent experiments. NS means non-specific bands. (**B, C, D**) Cell death was examined using a double staining apoptosis assay (Hoechst33342/PI) (**B**) and % PI-positive cells were presented in the graph. (**C**) Intracellular reactive oxygen species (ROS) and (**D**) mitochondrial ROS (mtROS) formation were examined by DCFDA and mitoSOX staining, respectively. Bar graph shows mean ± SD from at least 500 cells/condition compiled from three to six experiments. Scale bar represents 100 μm. (**E**) HeLa cells were pretreated with 10 μM mitoTEMPO (MT) for 24 h prior to T/C or co-treated with T/C for 3 h. mtROS production was examined by mitoSOX staining. Bar graphs show mean ± SD of relative fluorescence intensity quantified by ImageJ. (**F**) HeLa cells were treated with identical conditions with (**A-D**) and examined by TMRM assay. 25 μM carbonyl cyanide phenylhydrazone (CCCP) was used as a positive control as a mitochondrial depolarizing agent. *p < 0.05; **p < 0.01 vs. T/C-treated cells, ##*p* < 0.01; ###*p* < 0.001 vs. Ctl-treated cells.

TNF-α is a proinflammatory cytokine and a well-known modulator of cell survival and cell death [17]. Even though TNF-α treatment alone cannot induce apoptosis in vitro, combined treatment with TNF-α and CHX can cause apoptosis by ROS production [18]. At first, we investigated whether treatment with the oxidative stress-causing agent AS can suppress TNF-α/CHX-mediated apoptosis upon pre- or co-treatment with TNF-α/CHX in HeLa cells. The expression levels of cleaved PARP, cleaved-caspase-8, and cleaved-caspase-3/7 were significantly reduced, although cleavage of caspase-9, a marker of intrinsic apoptosis, was not observed upon TNF-α/CHX stimulation (Fig. 1A). Next, we conducted a double staining apoptosis assay (Hoechst33342/PI) to measure cell death. PI-positive cells were abundant in TNF-α/CHX-stimulated cells, but pre- or co-AS treatment resulted in significantly lower numbers of dead cells (Fig. 1B). To determine the mechanisms whereby SG induction protected against apoptosis, we explored the level of ROS production following AS treatment. Most cells were found to produce ROS in the TNF-α/CHX-treated condition, and we found that AS treatment significantly suppressed ROS production. Consistent with DCFDA staining, mitoSOX staining, as an indicator of the superoxide production specific in mitochondria, indicated the reduction of mitochondria-specific ROS production by AS treatment (Figs. 1C and 1D). Furthermore, we examined whether the mitochondria-specific ROS scavenger mitoTEMPO would reduce mitochondrial ROS and apoptosis. There was a significant downregulation of mitoSOX fluorescence by T/C in cells treated with mitoTEMPO (Fig. 1E). Additionally, mitochondrial membrane potential (MMP), which is a key parameter of mitochondrial function and apoptosis, was determined by measuring the fluorescence intensity of tetramethyl-rhodamine methyl ester (TMRM). TNF-α/CHX treatment resulted in mitochondrial membrane depolarization, whereas pre- or co-treatment with AS resulted in upregulation of MMP level, suggesting that SGs were involved in modulating mitochondrial ROS production and function (Fig. 1F).

**Fig. 2:**
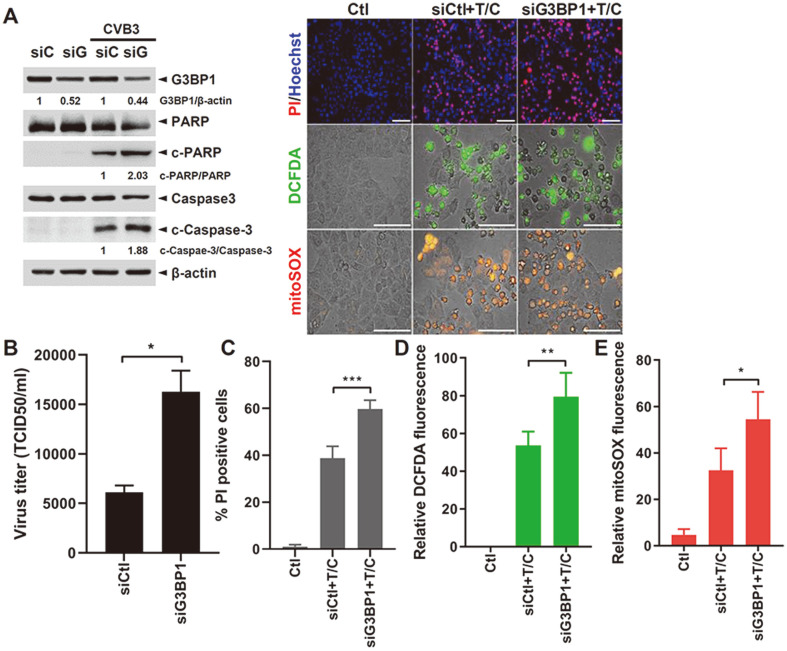
SG protein G3BP1 leads to control of apoptosis signaling pathways. (**A**) HeLa cells were either transfected with control siRNA (siC) or G3BP1-specific (siG) and then infected with CVB3-GFP at a multiplicity of infection of 1 for 8 h. The cleavage of caspase-3 and PARP is shown by Western blot analysis. Images are representatives of three independent experiments and band intensity was quantitated by densitometric analysis using ImageJ. (**B**) Supernatant was collected and viral titers were measured using the TCID50 assay and expressed as TCID50/ml (means ± SD; *n* = 3). *p < 0.05; vs. siCtltransfected cells. (**C**) HeLa cells were either transfected with control siRNA (siCtl) or G3BP1-specific (siG3BP1) and then treated with TNF-α and CHX (T/C) for 3 h. Cell death was examined using a double staining apoptosis assay (Hoechst33342/ PI) and representative images of PI-positive cells are shown. Bar graph shows mean ± SD from at least 500 cells/condition compiled from three experiments. PI-positive cells are indicated in red, and nuclei are stained blue. (**D**) Intracellular reactive oxygen species (ROS) and (**E**) mitochondrial ROS formation were examined by DCFDA and mitoSOX staining, respectively. Bar graphs show mean ± SD of relative fluorescence intensity quantified by ImageJ. Scale bar represents 100 μm. *p < 0.05; **p < 0.01; ***p < 0.001 vs. siCtl-transfected cells.

Next, to examine the role of G3BP1, a key SG component in apoptosis, we transfected G3BP1-specific siRNA into HeLa cells following CVB3-GFP infection or TNF-α/CHX treatment. G3BP1 is the most widely studied and commonly used marker for SGs and is reported to be an indispensable core protein of SG assembly induced by AS [19]. When G3BP1 expression was attenuated by gene knockdown, the levels of cleaved protein of PARP and the caspase-3 ratio were increased via the CVB3-mediated cell death pathway (Fig. 2A). Furthermore, CVB3 replication was found to be significantly increased upon G3BP1 knockdown, suggesting the antiviral role of G3BP1 (Fig. 2B). In accordance with the increase in apoptosis-related protein expression, double staining with Hoechst and PI showed increased numbers of PI-positive cells following knockdown of G3BP1 (Fig. 2C). Moreover, the levels of intracellular ROS and mitochondrial ROS increased with downregulated expression of G3BP1 (Figs. 2D and 2E). Taken together, G3BP1 is involved in inhibiting apoptotic cell death, possibly via suppressing mitochondrial ROS production.

### CVB3-Mediated Cell Death was Modulated by SG Formation Possibly via Mitochondrial ROS Reduction.

**Fig. 3:**
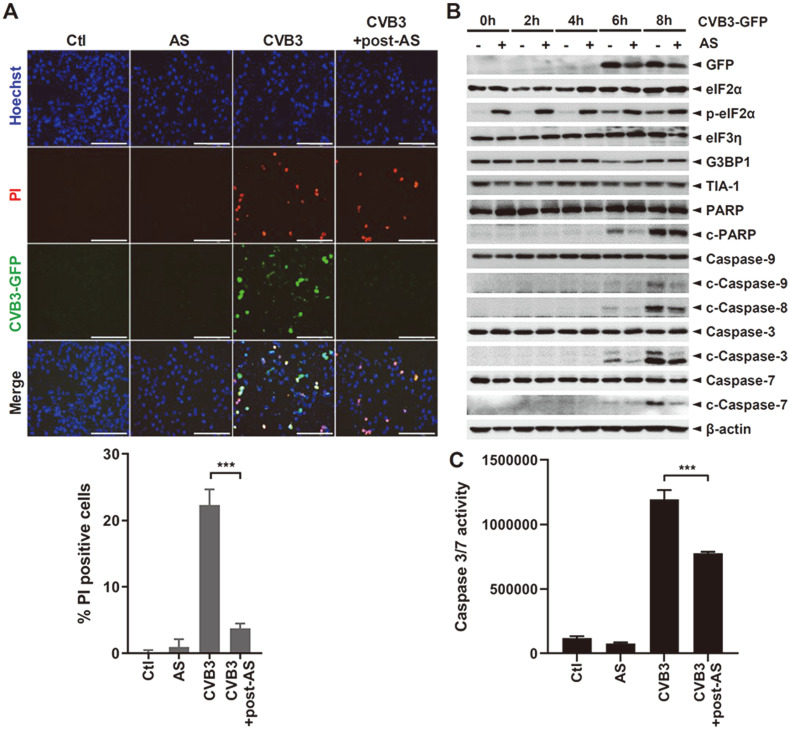
SGs inhibit CVB3-mediated cell death. (**A**) HeLa cells were either infected with CVB3 or treated with 0.5 mM sodium arsenite (AS) following infection for 0.5 h. Cell death was examined using the double staining apoptosis assay (Hoechst33342/PI) at 8 h post-infection, and representative images of PI-positive cells are shown. At least 500 cells were counted per experiment (*n* = 3). PI-positive cells are indicated in red, and nuclei are stained blue. Scale bar represents 200 μm. ***p < 0.001 vs. CVB3-infected cells. (**B**) HeLa cells were infected with CVB3-GFP for indicated time points. Cells were then treated with control (−) or 0.5 mM sodium arsenite (AS) (+) following infection. The cleavage of caspase-3, caspase-7, caspase- 8, caspase-9, and PARP is shown by Western blot analysis. Images are representatives of three independent experiments. (**C**) The caspase-3/7 activities of cells treated with control (Ctl) or 0.5 mM sodium arsenite (AS) were measured by caspase-Glo 3/7 activity assays. Data are represented as mean ± SD. ***p < 0.001 vs. CVB3-infected cells.

Given that CVB3 replication was inhibited by the accumulation of SGs at the early stage of viral infection [20], we next wanted to examine whether CVB3-induced cell death was inhibited by SG induction. Following AS treatment of CVB3-infected HeLa cells, the numbers of PI-stained cells as well as CVB3-infected cells were significantly diminished by SG induction (as shown in Fig. 3 by immunofluorescence staining), indicating that SGs impeded CVB3-mediated cell death. To explore the effect of SGs on apoptotic signaling pathways, cells were infected with CVB3 at various time points followed by AS post- treatment, and the apoptosis signaling pathways were examined by Western blotting (Fig. 3B). AS treatment initially suppressed CVB3 protein expression at 6 and 8 h post-infection. Expression of phospho-eIF2α was upregulated upon AS treatment while that of cleaved PARP and caspase 3, 7, 8, and 9 proteins were downregulated, suggesting the antiapoptotic role of SG formation. Consistent with this data, we observed similar results in the caspase 3/7 luminescence assays (Fig. 3C).

**Fig. 4:**
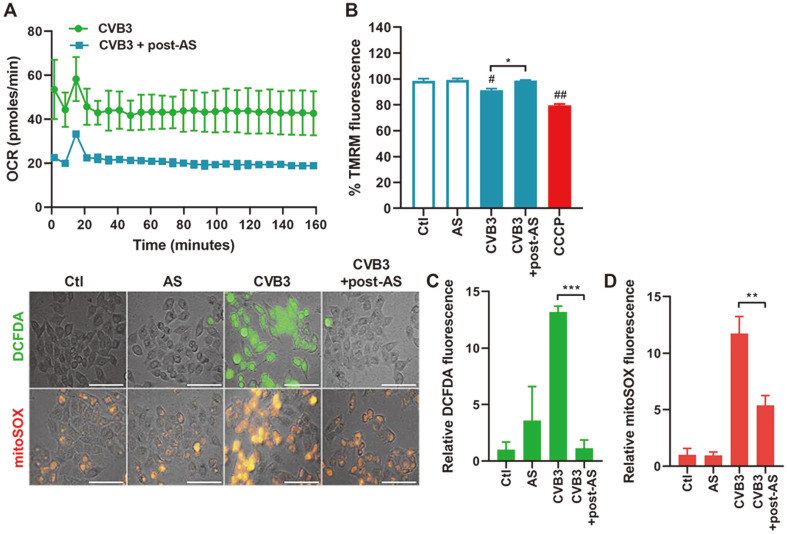
SGs inhibit CVB3-induced mitochondrial reactive oxygen species (ROS). (**A**) Mitochondrial respiration profile of HeLa cells infected with CVB3 at a multiplicity of infection (MOI) of 1 for 8 h or followed by 0.5 mM sodium arsenite (AS) treatment, is shown. The oxygen consumption rate (OCR) was measured over time (min) using a Seahorse XFp extracellular flux analyzer. (**B**) Mitochondrial membrane potential was measured by TMRM assay. CCCP treatment was used as a positive control. *p < 0.05 vs. CVB3-infected cells, #*p* < 0.05; ##*p* < 0.01 vs. Ctl-treated cells. (**C**) Intracellular ROS was examined by DCFDA staining. (**D**) Mitochondrial ROS was measured by mitoSOX staining. Scale bar represents 100 μm and bars graph show mean ± SD of fluorescence intensity quantified by ImageJ. **p < 0.01; ***p < 0.001 vs. CVB3-infected cells.

Previous studies indicate a close link between mitochondrial respiration profile and mitochondrial ROS production [21, 22]. Thus, we assessed the effect of SG formation on mitochondria bioenergetics and found that AS treatment in CVB3-infected cells resulted in downregulation of basal oxygen consumption rate (OCR) (Fig. 4A). Furthermore, AS treatment led to a significant change in MMP in response to CVB3 infection, similar to that of TNF-α/CHX treatment (Fig. 4B). We then sought to determine whether SG effect on mitochondrial membrane depolarization and programmed cell death activation was related to ROS production. AS treatment reduced ROS formation caused by CVB3-mediated cytopathic effect in HeLa cells at various time points (Fig. 4C). Similarly, mitoSOX staining revealed that mitochondrial ROS levels were significantly lowered by AS treatment (Fig. 4D).

### SGs Block the Extracellular Release of CVB3 via sEVs

**Fig. 5:**
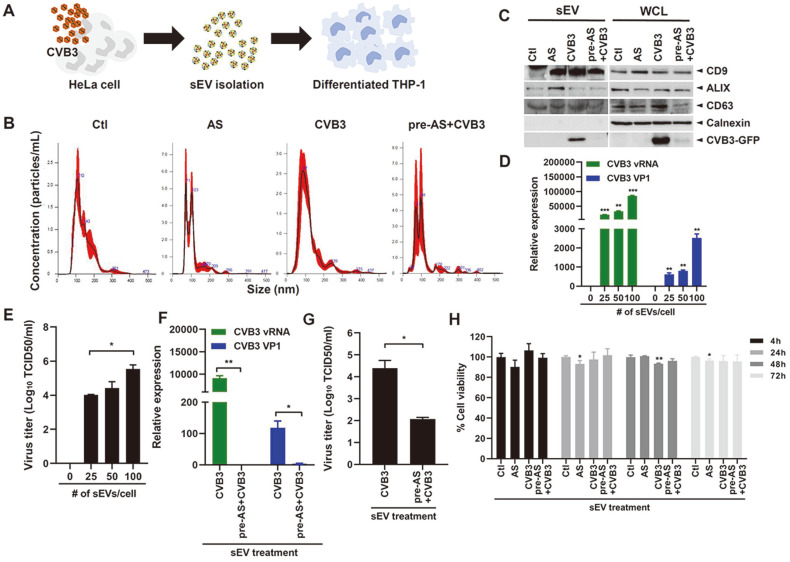
CVB3-enriched sEVs modulate THP-1 response, and SG activation inhibits extracellular release of CVB3 via sEVs. (**A**) Overview of experimental design. (**B**) HeLa cells were cultured in medium containing exosomedepleted FBS, treated with mock (Ctl) or 0.25 mM sodium arsenite (AS), or infected with CVB3-GFP (multiplicity of infection of 1) for 16 h with AS pre-treatment (pre-AS+CVB3) or without AS treatment (CVB3). Culture supernatant was collected and centrifuged at 300×*g* for 10min to remove cellular debris including apoptotic bodies. Subsequently, conditioned medium was collected, and small extracellular vesicles (sEVs) were isolated using Exo-Spin precipitation and size exclusion chromatography. Nanoparticle tracking analysis of sEVs shows that the mean diameter of the isolated sEVs was <150 nm. (**C**) Western blot analysis of sEV markers (CD9, CD63 and ALIX) is shown; calnexin (endoplasmic reticulum marker) was used as a negative control; GFP demonstrated CVB3. WCL: whole cell lysates (**D**) sEVs from CVB3 infected HeLa cells were added to THP-1 cells at various concentrations. qRT-PCR was performed to measure CVB3 RNA and VP1 relative expression levels. **p < 0.01; ***p < 0.001 vs. untreated cells. (**E**) Viral titers were measured using the TCID50 assay and expressed as log10TCID50/ml (means ± SD; *n* = 3). (**F**) sEVs from CVB3-infected cells with or without AS pre-treatment were added to THP-1 cells for 24 h. qRT-PCR was performed to measure CVB3 RNA and VP1 relative expression levels. Data are represented as mean ± SD. (**G**) Viral titers were measured using the TCID50 assay and expressed as log10TCID50/ml (means ± SD; *n* = 3). *p < 0.05 vs. cells treated with sEVs from CVB3-infected HeLa cells. (**H**) Cell viability was measured by CCK8 assay. Data are represented as mean ± SD. *p < 0.05; **p < 0.01 vs. ctl-treated cells.

Several viruses are known to spread from virus-infected cells to the intercellular space via packaging of virus particles into sEVs [23]. CVB3 is known to utilize vesicle-mediated transmission, and we therefore investigated whether SG formation affected CVB3 viral egress via sEVs. First, we isolated sEVs via precipitation and size exclusion chromatography and then measured the size and concentration of sEVs in each condition. SG formation in HeLa cells reduced mean size or distribution of sEVs, as demonstrated by nanoparticle tracking analysis (Figs. 5A and 5B). Next, sEV isolation was confirmed by measuring the expression of sEV markers, such as CD9, CD63 and ALIX, via Western blotting analysis (Fig. 5C). Isolated sEVs were positive for CD9, CD63, and ALIX, whereas calnexin was used as a negative control and was only detected in whole cell lysates, demonstrating that sEVs were not contaminated with cell debris. These sEVs were used in subsequent THP-1 stimulation experiments. Notably, CVB3-GFP expression was significantly impaired in sEVs from AS-treated cells, suggesting that SGs lower the vesicle-mediated CVB3 transmission.

To further examine whether CVB3-enriched sEVs can modulate the human monocytic cell (THP-1) response, we treated sEVs derived from CVB3-infected HeLa cells into THP-1 cells in a dose-dependent manner and evaluated CVB3 RNA and VP1 copy numbers using qRT-PCR (Fig. 5D). Viral gene expression was increased with addition of sEVs. In accordance with qRT-PCR, viral infectivity in THP-1 cells was increased in a dose-dependent manner with sEV treatment (Fig. 5E).

To determine the role of SGs in the process of sEV-mediated viral transmission, we evaluated viral replication and infectivity in THP-1 samples treated with sEVs isolated from CVB3-infected HeLa cells versus CVB3-infected HeLa cells treated with AS. Figs. 5F and 5G indicate that sEVs isolated from CVB3-infected cells treated with AS were able to suppress viral replication and infectivity in THP-1 cells, compared with viral infectivity induced by sEVs from untreated CVB3-infected cells, whereas sEV treatment did not cause significant changes in THP-1 viability (Fig. 5H). Therefore, these data suggest that CVB3-enriched sEVs modulate THP-1 cellular response and that SG activation blocks the release of CVB3 via sEVs.

## Discussion

SGs are typically assembled under acute or chronic stress conditions and enable transient translational arrest to prevent unnecessary protein synthesis or sequester malfunctioning misfolded proteins. In particular, the role of SGs during virus infection has been explored as an antiviral platform that recruits pattern recognition receptors, such as retinoic acid-inducible gene I (RIG-I) and double-strand RNA-activated protein kinase (PKR) [24]. Recent studies demonstrated that G3BP1, an important SG core protein, directly interacts with RIG-I via the c-terminal domain and enhances RIG-I-induced IFN-b gene expression. Furthermore, overexpression of G3BP1 suppressed viral replication during Sendai virus and Vesicular stomatitis virus infection [25, 26]. SG formation is modulated during CVB3 infection, and the enteroviral proteolytic enzyme protein 2A has a conserved role in inhibiting innate antiviral responses by suppressing SG assembly and IFN-b gene transcription [27].

SGs can delay stress-induced cell death via sequestering of several apoptosis regulatory factors such as receptor for activated C kinase 1 (RACK1) and the regulatory-associated protein in mammalian target of rapamycin complex 1 (mTORC1) [28, 29]. Similarly, we investigated whether TNF-α/CHX-induced apoptosis can be modulated by SG induction. AS-mediated SG formation was found to suppress cell death induced by TNF-α/CHX treatment, during which G3BP1, an essential SG component, inhibited apoptotic cell death. Hence, it would be interesting to further examine whether G3BP1 interacts with any of apoptosis regulatory factors.

ROS are byproducts of oxygen metabolism that can mediate cell death. Superoxide (O_2_^−^) generated in the mitochondria is the main source of intracellular ROS and is a known indicator of mitochondrial ROS [30], which is upregulated during virus infection and is beneficial for viral replication as a survival strategy. Inhibition of mitochondrial ROS production using antioxidant treatment impedes virus infection and virus-induced cell death [6, 31-34]. Given that AS-mediated SG formation suppresses ROS production and ROS-induced apoptosis via G3BP1 and ubiquitin-specific protease 10 [35], we investigated whether ROS and mitochondrial ROS were modulated by SG formation during CVB3 infection. SG activation not only reduced CVB3 replication but also that of CVB3-induced apoptosis. First, we confirmed that SG induction promotes antiviral function against CVB3 infection and antiapoptotic function given the reduction in levels of activated caspase-3/7, caspase-8, caspase-9, and PARP. Additionally, CVB3-induced apoptotic cells were significantly reduced upon AS-mediated SG formation, possibly via the reduction of mitochondrial ROS. Since AS-mediated SG formation led to the alteration of OCR in cells, it is possible that SG aggregates can affect the mitochondrial metabolic function of CVB3-infected cells.

CVB3 is known to be released via sEVs as an immune escape mechanism formed by intracellular autophagic flux, and thus sEV-mediated viral egress allows viruses to exit from infected cells [11]. EV-contained virus inoculation enhances viral infectivity compared with that produced by free virus inoculation, enabling recipient cells to adopt virus particles clustered in EVs [32]. In our study, we investigated whether SG induction inhibited extracellular release of CVB3 using AS-treated HeLa cells infected with CVB3 from which sEVs were isolated and then added to THP-1 cells for subsequent analysis. Use of SG-induced sEVs lowered viral replication and infectivity compared with that in cells treated with CVB3-enriched sEVs, although no changes occurred in cell viability. These findings suggest that SG induction may inhibit the mechanism of sEV-mediated extracellular release of CVB3. Further studies on the interplay between SGs and sEV formation will facilitate understanding of the potential role of SG formation in sEV biogenesis and secretion.

While the role of SGs during cellular stress is generally cytoprotective, the interaction between SGs and virus-mediated apoptosis has not been clearly demonstrated. In this study, we provided evidence of a potential mechanism whereby SG formation may inhibit apoptosis via reduction of mitochondrial ROS production and extracellular release of CVB3 via sEVs. It will be interesting to further investigate the potential of specific SG inducers or inhibitors to modulate disease pathologies associated with apoptosis.
